# Supplementary material for: Galectin-3 impairs calcium transients and β-cell function
Source: Nat Commun. 2024 May 1;15:3682. doi: 10.1038/s41467-024-47959-1 (PMC11063191; doi:10.1038/s41467-024-47959-1)
Supplement: Supplementary file 1 — Supplementary Information [file 41467_2024_47959_MOESM1_ESM.pdf]

Supplementary information

Galectin-3 Impairs Calcium Transients and β-Cell Function

Qian Jiang[1,2,3,10], Qijin Zhao[1,2,3,10], Yibing Chen[1,2,3,10], Chunxiao Ma[1,2,3,10], Xiaohong Peng[4,5], Xi Wu[6,7], Xingfeng Liu[1,2,3], Ruoran Wang[8], Shaocong Hou[1,2,3], Lijuan Kong[1,2,3], Yanjun Wan[1,2,3], Shusen Wang[9], Zhuo-Xian Meng[8], Bing Cui[1,2,3], Liangyi Chen[4,5,7], Pingping Li[1,2,3*]

Supplementary figures

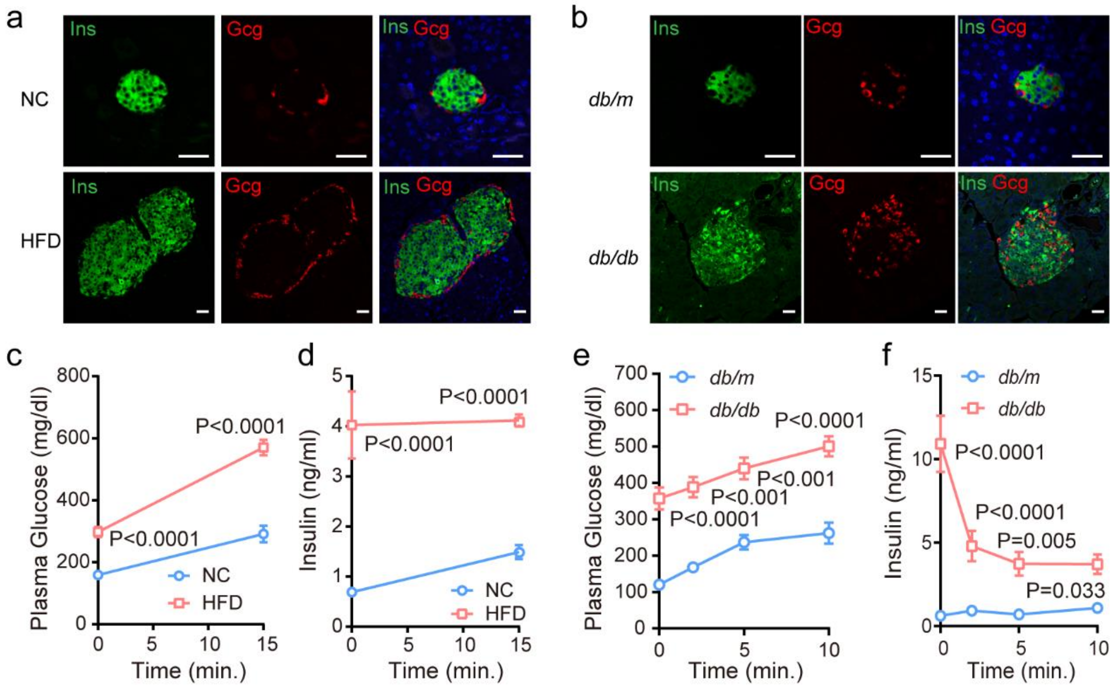

Supplementary Fig. 1 β-cell function was decreased in HFD-fed and *db/db* mice. a, Immunohistochemistry analysis of insulin (green) and glucagon (red) in pancreas of NC and HFD-fed mice. **b,** Immunohistochemistry analysis of insulin (green) and glucagon (red) in pancreas of *db/m* and *db/db* mice. **c-d,** IPGTT (**c,** n = 5 mice) and first-phase insulin secretion (**d,** n = 7 mice) in NC and HFD-fed mice after 6 h of fasting. **e-f,** IPGTT (**e**) and first-phase insulin secretion (**f**) in *db/m* and *db/db* mice after 6 h of fasting. Data were analyzed by two-sided Student's *t*-test without adjustments for multiple comparisons. All data are presented as the mean ± SEM. Source data are provided as Source Data file. n = 6 islets in 4 pancreatic sections (**a-b**), n = 5 *db/m* mice, n = 9 *db/m* mice (**e-f**). NC mice: 20 weeks of age, HFD: 12 weeks feeding from 8 weeks of age; *db/m* and *db/db* mice: 28 weeks of age (**a-f**). Scale bar, 20 μm.

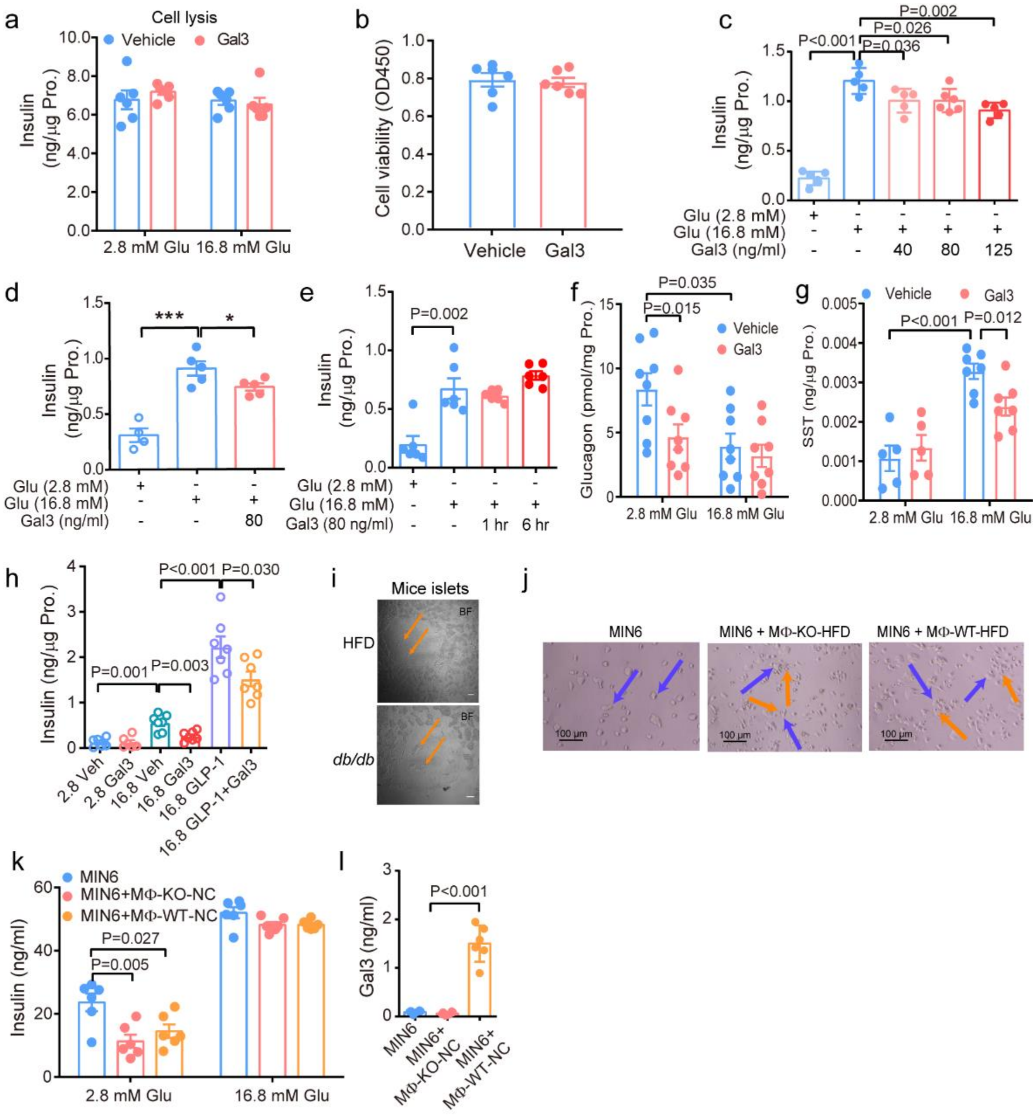

Supplementary Fig. 2 MIN6 cells coculture with MΦ from NCD mice. a, Insulin content in MIN6 cells during GSIS with Gal3 (80 ng/ml, 6 h) treatment. **b,** Cell viability of MIN6 cells with vehicle or Gal3 (125 ng/ml, 6 h) treatment. **c,** GSIS in MIN6 cells with Gal3 (40, 80, 125 ng/ml, 6 h) treatment. **d,** GSIS in MIN6 cells with Gal3 (80 ng/ml, 1 h) treatment. **e,** Effect of Gal3 (80 ng/ml) on GSIS in MIN6 cells with Gal3 treatment in DMEM culture medium for 1 h or 6 h, followed by washing twice with 2.8 mM KRH buffer and performing GSIS without Gal3. **f-g,** Glucagon (**f**) and somatostatin (**g**) secretion with Gal3 treatment (250 ng/ml, 1 h) in mice islets in the process of GSIS. **h,** GLP-1 (100 nM) stimulated insulin secretion in mice islets with Gal3 (250 ng/ml, 1 h) treatment. **i,** Bright field of HFD-fed and *db/db* mice islets. The yellow arrow points to MΦ. **j,** Bright field of MIN6 cells and MΦ coculture system. The blue arrow points to MIN6 cells, the yellow arrow points to MΦ. The MΦ were

peritoneal macrophages from WT or Gal3 KO mice on HFD feeding. **k,** GSIS in coculture system of MIN6 cells and peritoneal macrophages from WT and Gal3 KO mice on NC-fed. **l,** Gal3 levels in condition medium from coculture system in (**k**). Data were analyzed by two-sided Student's *t*-test without adjustments for multiple comparisons. All data are presented as the mean ± SEM. Source data are provided as Source Data file. n=6 (**a-b, e, h, k-l**), n=5 (**c-d**), n=8 (**f**), n=7 (**g**) biologically independent cell samples. n=3 independent experiment (**i, j**). Islets were isolated from 12-week-old NC mice, MΦ were isolated from 20-week-old NC mice or 12 weeks of HFD-fed mice from 8 weeks of age.

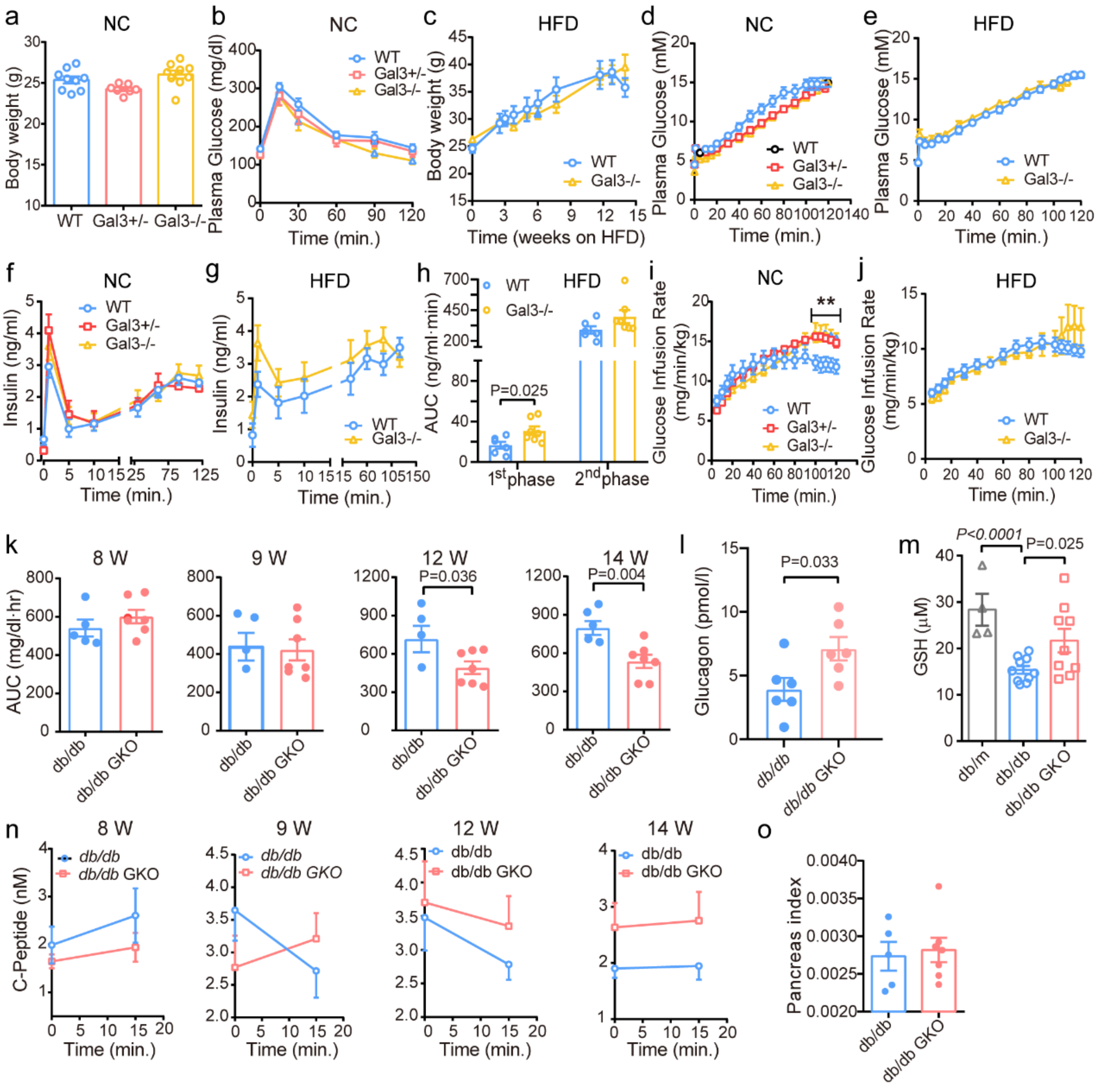

Supplementary Fig. 3 Metabolic studies in NC, HFD-fed and *db/db* Gal3 KO Mice. a-b, Body weight (**a**), and IPGTT (**b**) of WT, Gal3+/- and Gal3-/- mice on NC-fed. **c,**

Body weight in HFD-fed WT and Gal3-/- mice. **d-e,** Plasma glucose during hyperglycemic clamp study in WT, Gal3+/- and Gal3-/- mice on NC-fed (**d**) and in HFD-fed WT and Gal3-/- mice (**e**). **f,** Insulin level during hyperglycemic clamp study in WT, Gal3+/- and Gal3-/- mice on NC-fed. **g-h,** Insulin level (**g**) and the AUC of the first phase of insulin secretion (from 0 to 10 min) and the second insulin secretion (from 10 to 120 min) (**h**) during hyperglycemic clamp study in HFD-fed WT and Gal3-/- mice. **i-j,** Glucose infusion rate (GIR) during hyperglycemic clamp study in WT, Gal3+/- and Gal3-/- mice on NC (**i**) and in HFD-fed WT and Gal3-/- mice (**j**). **k**, The AUC of IPGTT in *db/db* and *db/db*-Gal3 KO mice (from 8 to 14 weeks of age). **l-m,** Plasma glucagon levels (**l**) and plasma glutathione (GSH) levels (**m**) in *db/db* and *db/db*-Gal3 KO mice (17 weeks of age). **n,** Plasma C-peptide concentration in *db/db* and *db/db*-Gal3 KO mice (from 8 to 14 weeks of age). **o,** Pancreas mass index of *db/db* and Gal3 KO mice (17 weeks of age). Data were analyzed by two-sided Student's *t*-test without adjustments for multiple comparisons. All data are presented as the mean ± SEM. Source data are provided as Source Data file. n = 7 Gal3+/- mice, n=8 Gal3-/- mice, n=9 WT mice (**a-b, d, f, i**); n=10 Gal3-/- mice, n=12 WT mice (**c**); n = 6 WT mice, n=8 Gal3-/- mice (**e, g-h, j**); n=5 *db/db* mice, n=7 *db/db*-GKO mice (**k, o**); n=6 mice (**l**); n=4 *db/m* mice, n=9 *db/db*-GKO mice, n=10 *db/db* mice (**m**); n=4 *db/db* mice, n=7 *db/db*-GKO mice (**n**). NC mice: 12 weeks old, HFD: 8-12 weeks feeding from 8 weeks of age. P value (**i**): 100 min. p=0.008, 105 min. p=0.006, 110 min. p=0.008, 115 min. p=0.010, 120 min. p=0.018. **p < 0.01.

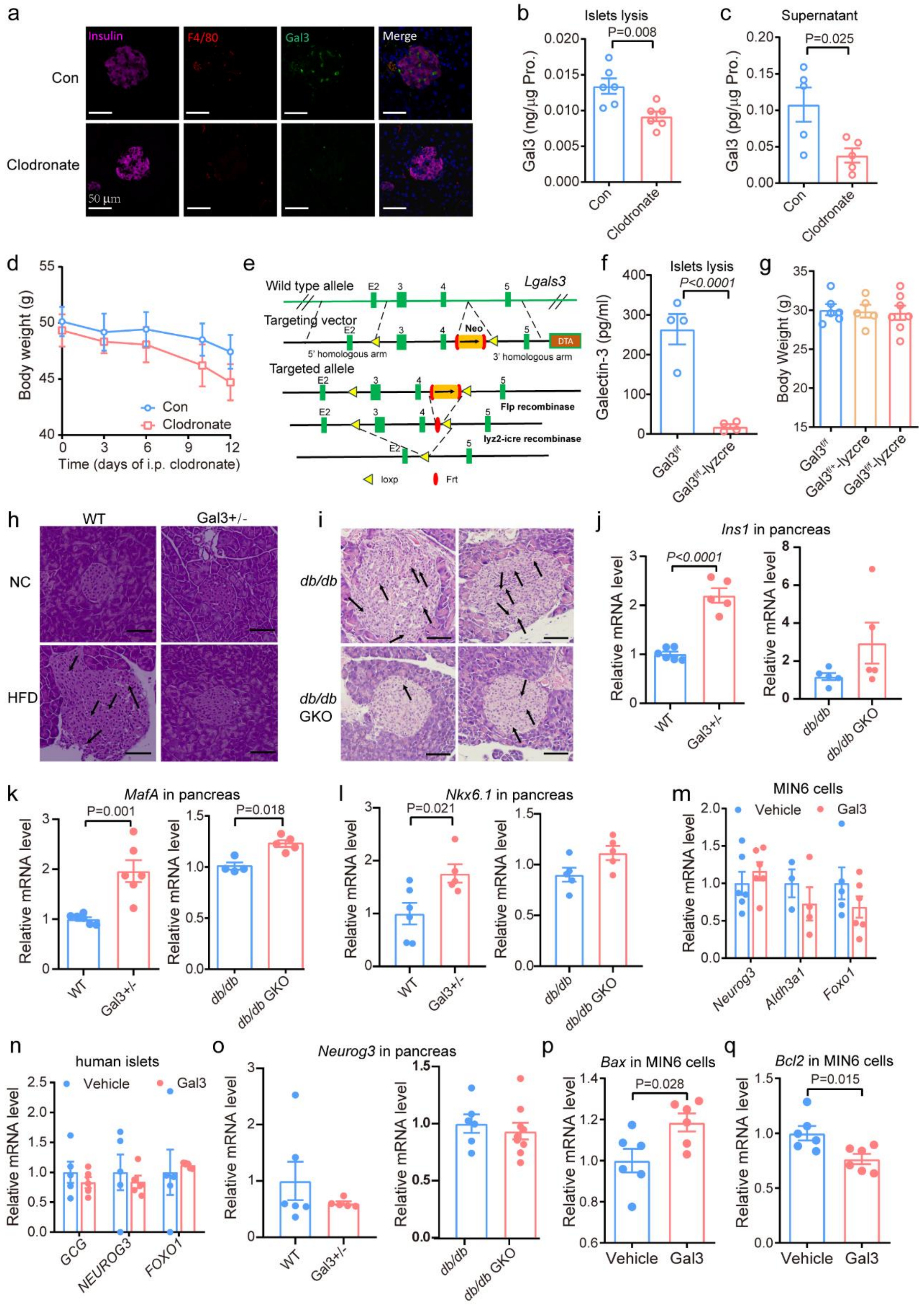

Supplementary Fig. 4 Gene expression studies about dedifferentiation and inflammation. a, immunofluorescence staining of F4/80 and Gal3 in pancreatic islets, n=5 islets in 5 pancreatic sections, Scale bar, 50 μm. **b-c**, Gal3 levels in islet (**b**) and

supernatant of islet (**c**) from clodronate- or control-treated HFD mice. **d**, Body weight. **e**, Schematic representation of conditional floxed and deleted *Lgals3* gene loci. **f**, Gal3 levels in islets from HFD-fed mice. g, Body weight of HFD-fed mice. HFD: 4 weeks feeding from 8 weeks old. **h-i,** HE staining of pancreatic islets from Gal3+/- (**h,** n=5 islets in 4 pancreatic section) and *db/db* mice (**i,** n=6 islets in 4 pancreatic section). The black arrow points to vacuolar degeneration cell. Scale bar, 100 μm. **j-l,** mRNA level of *Ins1* (**j**), *Mafa* (**k**) and *Nkx6.1* (**l**) in pancreas from Gal3+/- mice on HFD-fed and *db/db* GKO mice. **m,** mRNA level of genes involved in dedifferentiation in MIN6 cells treated with or without Gal3 (80 ng/ml, 24 h). **n,** mRNA level of *GCG*, *NEUROG3* and *FOXO1* in human islets treated with or without Gal3 (250 ng/ml, 24 h). **o,** mRNA level of *Neurog3* in pancreas of HFD-fed WT, Gal3+/- mice and *db/db, db/db* GKO mice. **p-q,** mRNA level of proapoptotic gene *Bax* (**p**) and anti-apoptotic gene *Bcl2* (**q**) in MIN6 cells treated with or without Gal3 (80 ng/ml, 48 h). HFD (**h, g-l, o**): 16 weeks feeding from 8 weeks old, *db/db* and *db/db* GKO: 16 weeks old. Data were analyzed by two-sided Student's *t*-test without adjustments for multiple comparisons. All data are presented as the mean ± SEM. Source data are provided as Source Data file. n = 6 biologically independent samples (**b-d, m-n, p-q**); n=4 biologically independent samples (**f**); n=6 mice ($Gal3^{f/f}$ and $Gal3^{f/f}$ -lyzcre), n=7 mice ($Gal3^{f/+}$ -lyzcre) (**g**); n=5 mice (**j-l, o**).

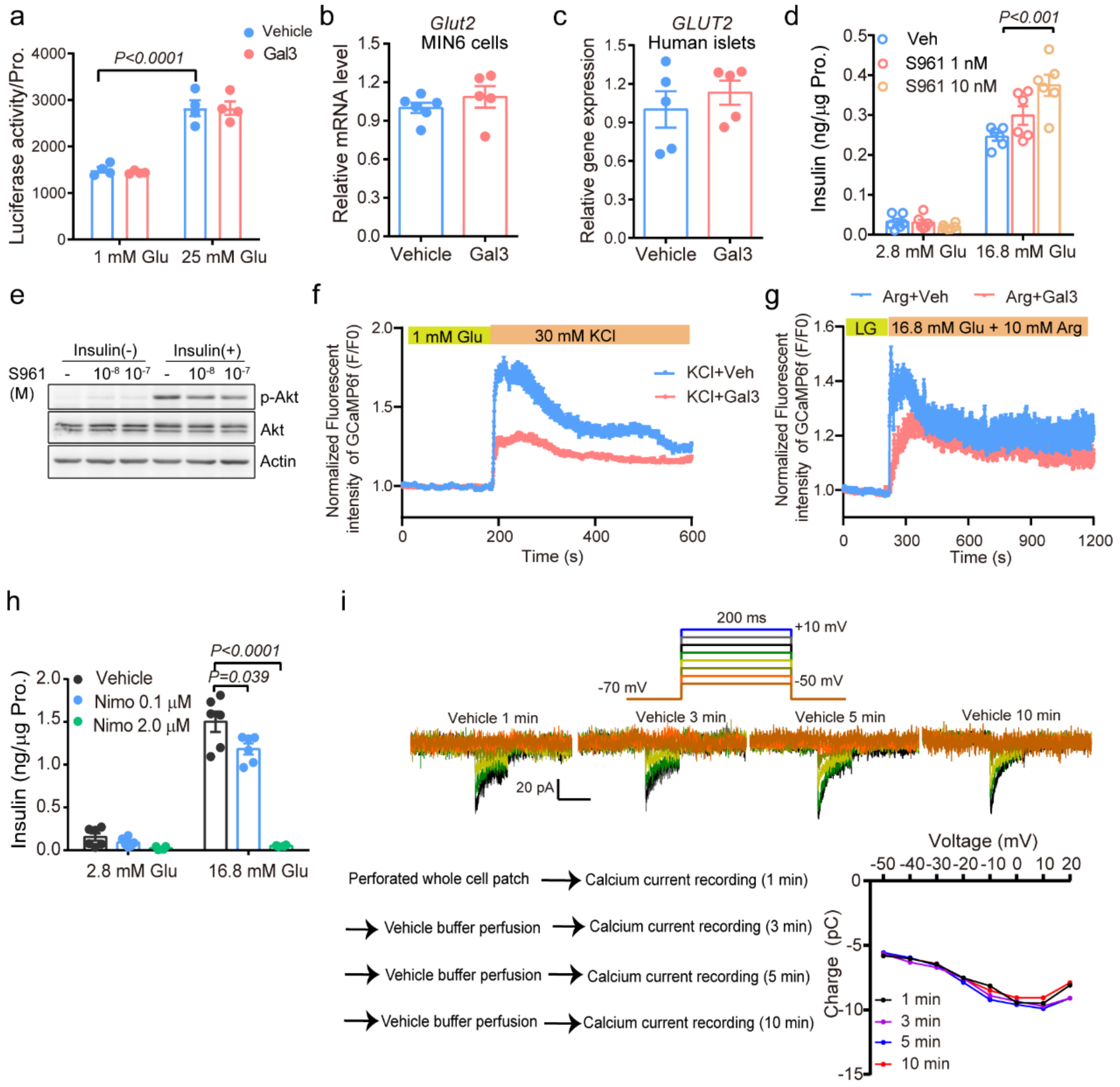

Supplementary Fig. 5 The effects of Gal3 on glucose uptake and insulin signaling pathway in β cells. a, Effect of Gal3 (80 ng/ml, 1 h) on glucose uptake in MIN6 cells. **b-c,** mRNA levels of *Glut2* genes in MIN6 cells (**b**) and islets from healthy persons (**c**) with or without Gal3 (80 ng/ml, 24 h). **d,** GSIS in MIN6 cells with S961 (1 nM and 10 nM, 1 h) treatment. **e,** Protein levels of AKT and phosphorylation-AKT in HepG2 cells with or without S961 (10^{-8} M and10^{-7} M, 6 h) or insulin (10 nM, 30 min). **f,** Typical traces of time-lapse calcium imaging in islets with KCl (30 mM) stimulation and Gal3 (250 ng/ml, 1 h) treatment. **g**, Typical traces of time-lapse calcium imaging in islets with arginine (10 mM) stimulation and Gal3 (250 ng/ml, 1 h) treatment. **h,** GSIS in MIN6 cells with Nimodipine (0.1 μM and 2 μM, 1 h) treatment. **i**, Ca^{2+} current records at a series of point time (1, 3, 5, and 10 min) in single MIN6 cell continuously perfused

with vehicle solution. Data were analyzed by two-sided Student's *t*-test without adjustments for multiple comparisons. All data are presented as the mean ± SEM. Source data are provided as Source Data file. n=4 (**a**), n=5 (**b-c**), n=6 (**d**) biologically independent samples; n = 23 β cells (**f**); n = 16 β cells (**g**). Islets were isolated from 12-week-old NC mice.

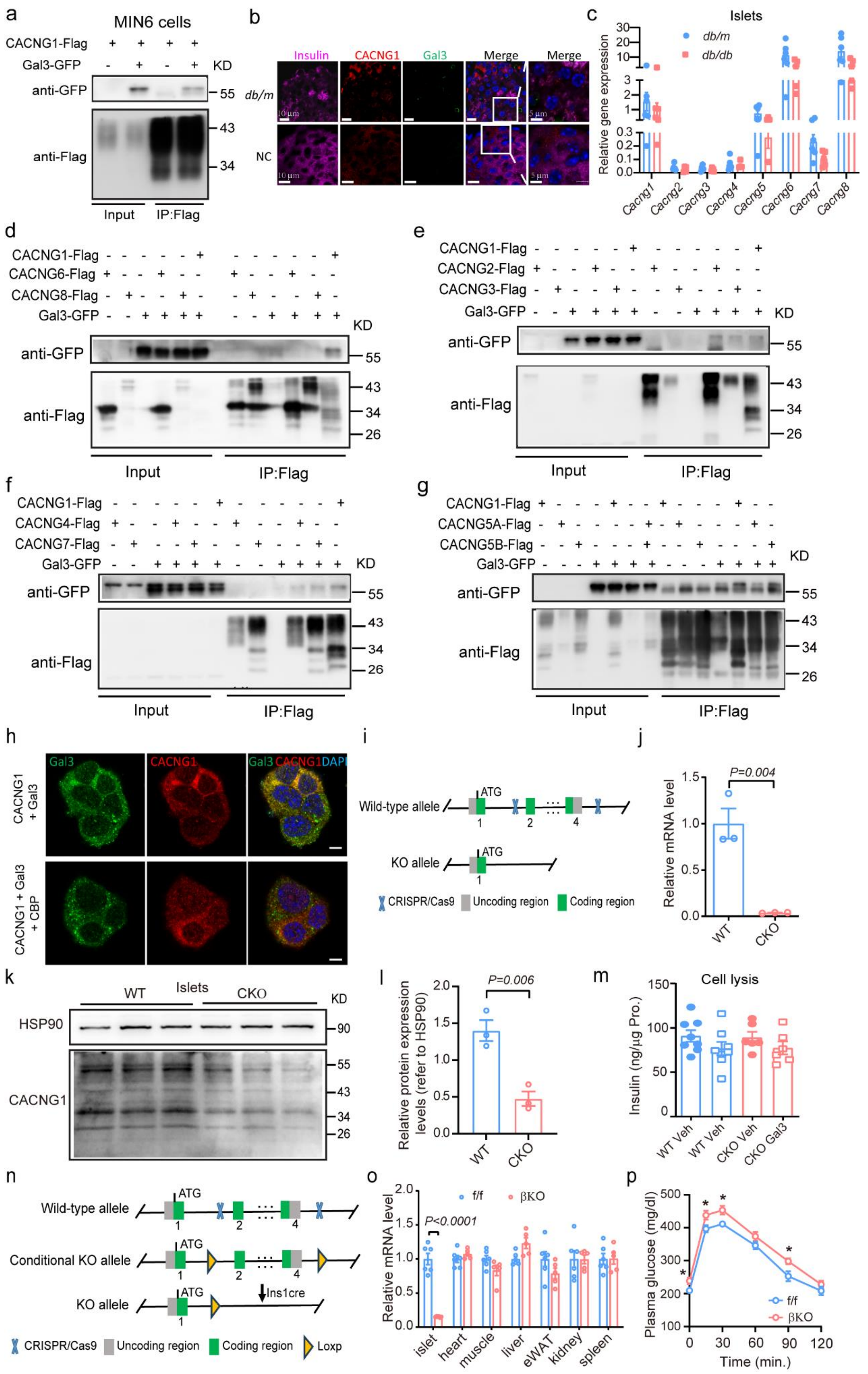

a
MIN6 cells
CACNG1-Flag
Gal3-GFP
KD
anti-GFP
anti-Flag
55
43
34
Input
IP:Flag
b
Insulin
CACNG1
Gal3
Merge
Merge
db/m
NC
10 μm
5 μm
c
Islets
db/m
db/db
Relative gene expression
Cacng1
Cacng2
Cacng3
Cacng4
Cacng5
Cacng6
Cacng7
Cacng8
d
CACNG1-Flag
CACNG6-Flag
CACNG8-Flag
Gal3-GFP
KD
anti-GFP
anti-Flag
55
43
34
26
Input
IP:Flag
e
CACNG1-Flag
CACNG2-Flag
CACNG3-Flag
Gal3-GFP
KD
anti-GFP
anti-Flag
Input
IP:Flag
f
CACNG1-Flag
CACNG4-Flag
CACNG7-Flag
Gal3-GFP
KD
anti-GFP
anti-Flag
Input
IP:Flag
g
CACNG1-Flag
CACNG5A-Flag
CACNG5B-Flag
Gal3-GFP
KD
anti-GFP
anti-Flag
Input
IP:Flag
h
Gal3
CACNG1
Gal3 CACNG1 DAPI
CACNG1 + Gal3
CACNG1 + Gal3 + CBP
i
Wild-type allele
ATG
KO allele
CRISPR/Cas9
Uncoding region
Coding region
j
P=0.004
Relative mRNA level
WT
CKO
k
WT
Islets
CKO
KD
HSP90
CACNG1
90
55
43
34
26
l
P=0.006
Relative protein expression levels (refer to HSP90)
WT
CKO
m
Cell lysis
Insulin (ng/μg Pro.)
WT Veh
WT Veh
CKO Veh
CKO Gal3
n
Wild-type allele
Conditional KO allele
KO allele
ATG
Ins1cre
CRISPR/Cas9
Uncoding region
Coding region
Loxp
o
f/f
βKO
P<0.0001
Relative mRNA level
islet
heart
muscle
liver
eWAT
kidney
spleen
p
Plasma glucose (mg/dl)
f/f
βKO
Time (min.)

Supplementary Fig. 6 Gal3 binding with CACNG1. a, Co-immunoprecipitation of Gal3-GFP and flag-tagged CACNG1 in MIN6 cells (n=2 independent experiments). **b,** Colocalization of Gal3 and CACNG1 was detected using antibodies against the endogenous proteins in islets from db/m and NC mice. (NC: 16 weeks old, C57BL6J, male; db/m: 17 weeks old, BKS, male). n=3 islets in 3 pancreatic section. **c,** mRNA levels of the *Cacng* family in islets of *db/db* mice, n=6 mice (12 weeks old). **d-g,** Co-immunoprecipitation of Gal3 and CACNG6, CACNG8 (**d**), CACNG2, CACNG3 (**e**), CACNG4, CACNG7 (**f**), CACNG5A, CACNG5B (**g**) in 293T cells. n=1 independent experiments (**d-g**). **h**, Confocal image of Gal3 colocalization with CACNG1 by double immunostaining in MIN6 cells. Scale bar, 5 µm. n=3 independent experiments. **i**, Schematic representation of WT and deleted *Cacng1* gene loci. **j,** *Cacng1* gene expression in islets from WT and *Cacng1* knockout (CKO) mice. **k-l,** CACNG1 protein levels in mouse islets from WT and CKO mice (n=3 biologically independent samples). **m,** Insulin content in islets during GSIS with or without Gal3 (250 ng/ml, 1 h) (n=8 WT, n=6 CKO biologically independent samples). **n**, Schematic representation of WT, conditional floxed and deleted *Cacng1* gene loci. **o**, *Cacng1* gene expression in tissues from *Cacng1*$^{f/f}$ and Cacng1$^{\beta KO}$ mice (n=6 mice). **p**, IPGTT of *Cacng1*$^{f/f}$ and Cacng1$^{\beta KO}$ mice (n=11 mice, HFD: feeding 4 weeks from 10 weeks of age. 0 min, P=0.024, 15 min, P=0.026, 30 min, P=0.012, 90 min, P=0.019). Data were analyzed by two-sided Student's *t*-test without adjustments for multiple comparisons. All data are presented as the mean ± SEM. Source data are provided as Source Data file. Mice were 10 weeks old (**j-m, o**). *$p < 0.05$.

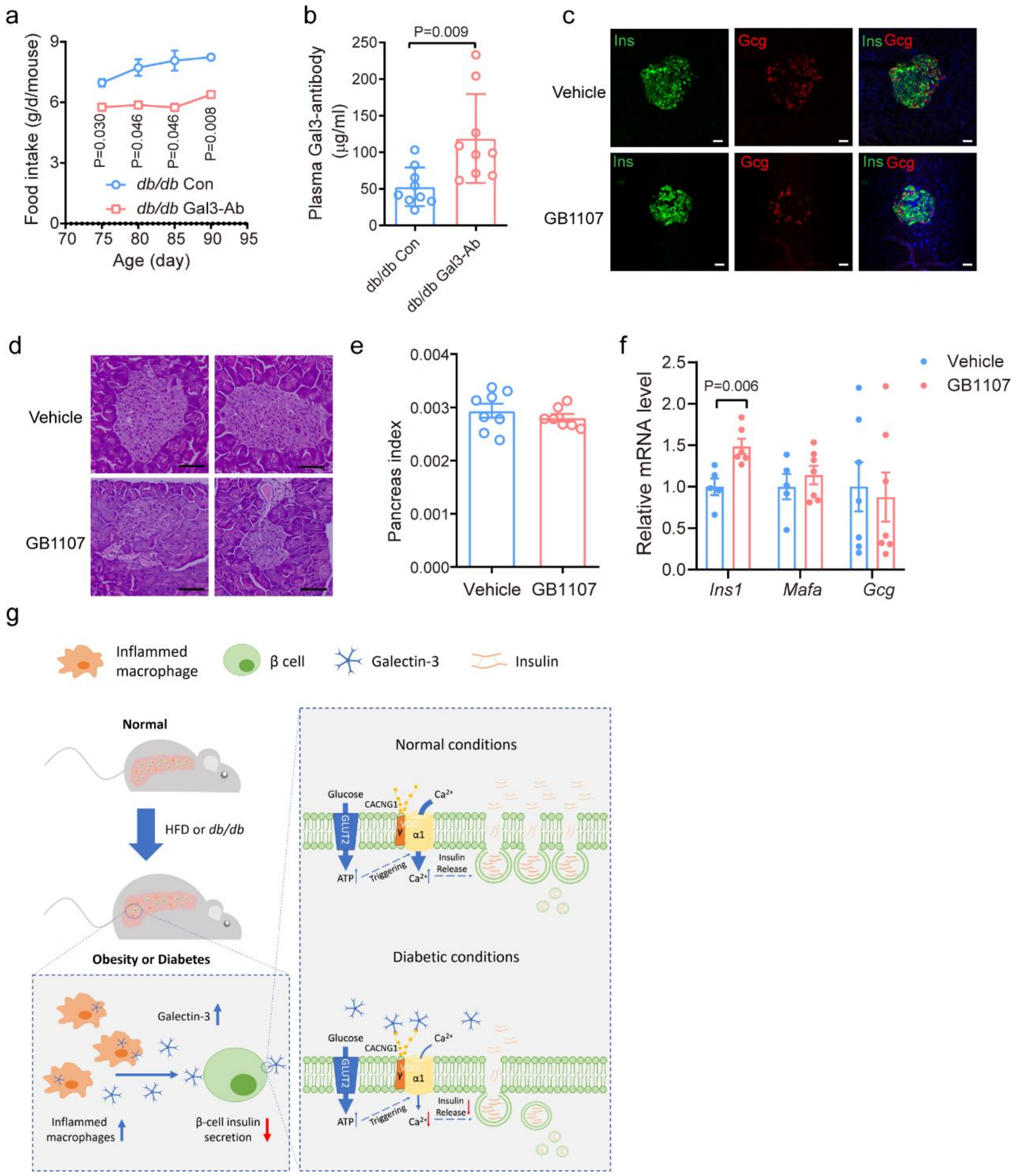

Supplementary Fig. 7 Histology and Gene Expression Studies of pancreas in *db/db* mice. a-b, Food intake (**a,** n=10 mice) and Blood Gal3 antibody levels (16 weeks of age) (**b,** n=9 mice) in *db/db* mice. **c,** Immunohistochemistry analysis of insulin (green) and glucagon (red) in pancreas of *db/db* mice. Scale bar, 20 μm. **d,** HE staining of pancreas from *db/db* mice. Scale bar, 100 μm. **e,** Pancreas mass index of *db/db* mice (n=8 mice). **f,** mRNA level of *Ins1*, *Mafa* and *Gcg* in pancreas from *db/db* mice. **g,** Graphic summary on Gal3 impairing calcium transients and β-cell function. Data were analyzed by two-sided Student's *t*-test without adjustments for multiple comparisons.

All data are presented as the mean ± SEM. Source data are provided as Source Data file. n = 5 islets in 5 pancreatic sections (**c-d**), n = 5 (*Ins1* and *Mafa*), n=7 (*Gcg*) biologically independent samples (**f**). Mice were 11 weeks old (**c-f**).

Supplementary tables

Supplementary Table 1 Key resources table

Reagent or Resource	Source	Identifier
Antibodies		
Rabbit anti-CD11c	Cell Signaling Technology	Cat# 97585
Guinea pig anti-insulin	Abcam	Cat# ab7842
Rat anti-insulin	R&D Systems	Cat# MAB1417
Rabbit anti-glucagon	Cell Signaling Technology	Cat# 2760
Mouse anti-galectin3	Abcam	Cat# ab2785
Rabbit anti-CACNG1	LSBio	Cat# LS-C805513
Rat anti-F4/80	Abcam	Cat# ab6640
Mouse anti-flag Tag	MBL	Cat# M185
Rabbit anti-GFP Tag	MBL	Cat# 598
Rabbit anti-Phospho S473 Akt	Cell Signaling Technology	Cat# 4060
Rabbit anti-Akt	Cell Signaling Technology	Cat# 4691
Mouse anti-beta actin	Proteintech	Cat# 66009-l-lg
Chemicals, Peptides, and Recombinant Proteins		
60% high-fat diet	Research Diets	Cat# D12492
Collagenase, Type V	Sigma-Aldrich	Cat# C9263
Galectin-3 protein	R&D Systems	Cat# 1197-GA-050
Galectin-3-GFP protein	This paper	N/A
L-Arginine	Sigma-Aldrich	Cat# A5131
GLP-1 (7-36)	MCE	Cat# HY-P0054
TRIzol RNA isolation reagent	Thermo Fisher Scientific	Cat# 15596026
RNA purification kit	QIAGEN	Cat# 74104
iTaq SYBR Green supermix	Bio-Rad Laboratories	Cat# 1725121
High-capacity cDNA reverse transcription kit	Thermo Fisher Scientific	Cat# 4368813
GB1107	MCE	Cat# HY114409
TD139	Selleckchem	Cat# S0471
Galectin-3 inhibitor Cpd47	Merck	N/A
Cacng1 blocking peptide	Alomone labs	Cat# BLP-CC111
Nimodipine	Sigma-Aldrich	Cat# N149
S961	Phoenix Pharmaceuticals	Cat# 051-86

Clodronate	Yeasen Biotechnology (Shanghai) Co., Ltd.	Cat# 40337ES10
Critical Commercial Assays		
Mouse insulin ELISA kit	ALPCO	Cat# 80-INSMSU-E10
Rat insulin ELISA kit	ALPCO	Cat# 80-INSRTU-E10
Human insulin ELISA kit	Mercodia	Cat# 10-1132-01
Mouse glucagon ELISA kit	EASYBIO	Cat# 10-1281-01
Mouse somatostatin ELISA kit	Phoenix Pharmaceuticals, Inc.	Cat# EK-060-03
Mouse C-Peptide ELISA kit	ALPCO	Cat# 80-CPTMS-E01
Mouse Galectin-3 ELISA kit	Aviscera Bioscience	Cat# SK00199-03
ATP ELISA kit	Promega	Cat# FF2000
Glucose Uptake-Glo™ Assay	Promega	Cat# J1343
Cell Counting Kit-8	DOJINDO	Cat# CK17
GSH Kit	Beyotime	Cat# S0053
Experimental Models: Cell Lines		
MIN6	Gift from Dr. Xu in Institute of Biophysics, Chinese Academy of Sciences	N/A
INS1	ACCEGEN	ABC-TC232S
HEK293T	ATCC	N/A
Human Pancreatic Islets	Tianjin First Central Hospital	N/A
Mouse Pancreatic Islets	This paper	N/A
Experimental Models: Organisms/Strains		
C57BL6/J	Shanghai Research Center For Model Organisms	
Gal3 KO mice	This paper	
db/db-Gal3 KO mice	This paper	
db/db mice	GemPharmatech Co，Ltd	
Recombinant DNA		
cacng1 c-myc plasmid	Origene	Cat# MR220452
cacng5 tv1 c-myc DDK mouse plasmid	Origene	Cat# MR228935
cacng5 tv2 c-myc DDK mouse plasmid	Origene	Cat# MR219914
Software and Algorithms		
ImageJ	NIH	https://imafej.nih.gov/ij/

Prism	Graphpad	https://www.graphpad.com

Supplementary Table 2 Primer sequences for mouse genes qPCR. Related to Fig. 1, Fig. 2 and Supplementary Fig.4.

**gene**	**Forward primer (5'-3')**	**Reverse primer (5'-3')**
Lgals3	AGACAGCTTTTCGCTTAACGA	GGGTAGGCACTAGGAGGAGC
Adgre1	CTTTGGCTATGGGCTTCCAGTC	GCAAGGAGGACAGAGTTTATCGTG
Itgax	CTGGATAGCCTTTCTTCTGCTG	GCACACTGTGTCCGAACTCA
Ccl2	TAAAAACCTGGATCGGAACCAAA	GCATTAGCTTCAGATTTACGGGT
Ccl5	TTTGCCTACCTCTCCCTCG	CGACTGCAAGATTGGAGCACT
Il6	CTGCAAGAGACTTCCATCCAG	AGTGGTATAGACAGGTCTGTTGG
Tnfα	CAGGCGGTGCCTATGTCTC	CGATCACCCCGAAGTTCAGTAG
Il-1β	TGGCAACTGTTCCTGAACTCAA	AGCAGCCCTTCATCTTTTGG
Il10	GCCAAGCCTTATCGGAAATG	CACCCAGGGAATTCAAATGC
Arg1	CTCCAAGCCAAAGTCCTTAGAG	AGGAGCTGTCATTAGGGACATC
Mrc1	CTCTGTTCAGCTATTGGACGC	CGGAATTTCTGGGATTCAGCTTC
Tgfb1	TAAAGAGGTCACCCGCGTGCTAAT	ACTGCTTCCCGAATGTCTGACGTA

Supplementary Table 3 Primer sequences for mouse genes qPCR. Related to Fig. 4 and Supplementary Fig. 4.

**gene**	**Forward primer (5'-3')**	**Reverse primer (5'-3')**
Ins1	GCTTCTTCTACACACCCATGTC	AGCACTGATCTACAATGCCAC
Mafa	CTGGAGGATCTGTACTGGATGA	CGCACGGACATGGATACCA
Nkx6.1	CTGCACAGTATGGCCGAGATG	CCGGGTTATGTGAGCCCAA
Neurog3	CCAAGAGCGAGTTGGCACT	CGGGCCATAGAAGCTGTGG
Foxo1	ATGCTCAATCCAGAGGGAGG	ACTCGCAGGCCACTTAGAAAA
Bax	TGAAGACAGGGGCCTTTTTG	AATTCGCCGGAGACACTCG
Bcl2	GTCGCTACCGTCGTGACTTC	CAGACATGCACCTACCCAGC
Glut2	TCAGAAGACAAGATCACCGGA	GCTGGTGTGACTGTAAGTGGG

Supplementary Table 4 Primer sequences for mouse genes qPCR. Related to Supplementary Fig. 6.

**gene**	**Forward primer (5'-3')**	**Reverse primer (5'-3')**
Cacng1	GCGCATTTCTGTCCTTCGGGAA	TACGCTTCACCGACTGCCTCAT
Cacng2	ACGAAGCTGACACCGCAGAGTA	TGGCGTGTCTTGTAGAACTCGC
Cacng3	CTTTCCGAGGCGTGTGCAAGAA	AGAGTGACGCTGAGGATGGGAA
Cacng4	GGAGGATCTACAGCCGCAAGAA	TCGCCCGTGTTGCTGGAAATGT
Cacng5	GCTCAACAGAACCAAGGATGCAG	CATGTCCTCAGCAGTGTACCTC
Cacng6	GTGGAAGGTGTGCATCAAGCGA	CTTGGTGGTTCGCTGGAAGATG

Cacng7	CAACTTGGTGACGGAAAACACGG	CGAACGCAAGAATGGTCCTCTG
Cacng8	CTCACAGCAGGTGATGACGGAC	GGTTGATCTTCACGCAGACACC

Supplementary Table 5 Primer sequences for human genes qPCR.

**gene**	**Forward primer (5'-3')**	**Reverse primer (5'-3')**
LGALS3	ATGGCAGACAATTTTTCGCTCC	GCCTGTCCAGGATAAGCCC
GCG	CTGAAGGGACCTTTACCAGTGA	CCTGGCGGCAAGATTATCAAG
NEUROG3	CTAAGAGCGAGTTGGCACTGA	GAGGTTGTGCATTCGATTGCG
FOXO1	TCGTCATAATCTGTCCCTACACA	CGGCTTCGGCTCTTAGCAAA
GLUT2	GCTGCTCAACTAATCACCATGC	TGGTCCCAATTTTGAAAACCCC

Supplementary Table 6 Information of donors for providing islets. Related to Fig. 7

Group	Age	Gender	BMI	HbA1c
Normal	20-30	Male	>24	NA
Normal	NA	NA	NA	NA
Diabetes	30-40	Male	>24	10
Normal	50-60	Male	>24	NA
Normal	40-50	Male	>24	5.3
Normal	50-60	Female	>24	5.8
Diabetes	40-50	Male	18.5-24	6.2
Normal	40-50	Male	18.5-24	5.3
Normal	40-50	Male	>24	5.4
Diabetes	50-60	Male	>24	6.7
Diabetes	50-60	Male	>24	7
Normal	40-50	Male	>24	5
Normal	40-50	Male	>24	5

NA: not available
