# Supplementary material for: Galectin-3 impairs calcium transients and β-cell function
Source: Nat Commun. 2024 May 1;15:3682. doi: 10.1038/s41467-024-47959-1 (PMC11063191; doi:10.1038/s41467-024-47959-1)
Supplement: Supplementary file 3 — Reporting Summary [file 41467_2024_47959_MOESM3_ESM.pdf]

nature portfolio

Corresponding author(s): Pingping Li

Last updated by author(s): Mar 25, 2024

Reporting Summary

Nature Portfolio wishes to improve the reproducibility of the work that we publish. This form provides structure for consistency and transparency in reporting. For further information on Nature Portfolio policies, see our Editorial Policies and the Editorial Policy Checklist.

Statistics

For all statistical analyses, confirm that the following items are present in the figure legend, table legend, main text, or Methods section.

n/a	Confirmed	
☐	☒	The exact sample size (*n*) for each experimental group/condition, given as a discrete number and unit of measurement
☐	☒	A statement on whether measurements were taken from distinct samples or whether the same sample was measured repeatedly
☐	☒	The statistical test(s) used AND whether they are one- or two-sided<br>*Only common tests should be described solely by name; describe more complex techniques in the Methods section.*
☒	☐	A description of all covariates tested
☒	☐	A description of any assumptions or corrections, such as tests of normality and adjustment for multiple comparisons
☐	☒	A full description of the statistical parameters including central tendency (e.g. means) or other basic estimates (e.g. regression coefficient) AND variation (e.g. standard deviation) or associated estimates of uncertainty (e.g. confidence intervals)
☐	☒	For null hypothesis testing, the test statistic (e.g. *F*, *t*, *r*) with confidence intervals, effect sizes, degrees of freedom and *P* value noted<br>*Give P values as exact values whenever suitable.*
☒	☐	For Bayesian analysis, information on the choice of priors and Markov chain Monte Carlo settings
☒	☐	For hierarchical and complex designs, identification of the appropriate level for tests and full reporting of outcomes
☒	☐	Estimates of effect sizes (e.g. Cohen's *d*, Pearson's *r*), indicating how they were calculated

Our web collection on statistics for biologists contains articles on many of the points above.

Software and code

Policy information about availability of computer code

Data collection	Standard software and the respective analysis tools provided by manufacturers were listed in the methods (Zeiss microsystems, QuantStudio 3, etc.). No software was used other than that listed in the Methods.
Data analysis	Student’s t-test analysis was done by using Excel and Prism GraphPad 6.0. All microscopy data was collected and analyzed using the Zeiss microsystems and ZEN software. Immunofluorescence and Western blots images analysis was performed by Image J.

For manuscripts utilizing custom algorithms or software that are central to the research but not yet described in published literature, software must be made available to editors and reviewers. We strongly encourage code deposition in a community repository (e.g. GitHub). See the Nature Portfolio guidelines for submitting code & software for further information.

Data

Policy information about availability of data

All manuscripts must include a data availability statement. This statement should provide the following information, where applicable:

- Accession codes, unique identifiers, or web links for publicly available datasets
- A description of any restrictions on data availability
- For clinical datasets or third party data, please ensure that the statement adheres to our policy

All data supporting the findings of this study are available from the corresponding authors upon request.

Research involving human participants, their data, or biological material

Policy information about studies with human participants or human data. See also policy information about sex, gender (identity/presentation), and sexual orientation and race, ethnicity and racism.

Reporting on sex and gender	We used human primary islets randomly from males and females. So these findings applied to either sex. The specific informations of donors for providing islets were provided in manuscript Supplementary Table 6.
Reporting on race, ethnicity, or other socially relevant groupings	The donors for providing islets were all Chinese.
Population characteristics	The specific informations of donors for providing islets were provided in manuscript Supplementary Table 6.
Recruitment	The human islets were all from organ donors with or without Type 2 diabetes disease and with research consents.
Ethics oversight	All protocols were approved by the Medical Ethical Committee of Tianjin First Central Hospital (No.2016N086KY).

Note that full information on the approval of the study protocol must also be provided in the manuscript.

Field-specific reporting

Please select the one below that is the best fit for your research. If you are not sure, read the appropriate sections before making your selection.

☒ Life sciences ☐ Behavioural & social sciences ☐ Ecological, evolutionary & environmental sciences

For a reference copy of the document with all sections, see nature.com/documents/nr-reporting-summary-flat.pdf

Life sciences study design

All studies must disclose on these points even when the disclosure is negative.

Sample size	Sample size was chosen as acceptable in the field of cell biology and animal experiments. Detailed description of the statistical methods used for the analyse, appears in the paper. Most of the in vitro experiments were repeated with at least three biological and/or technical replicates with similar results except for those specifically indicated in the figure legends. For in vivo experiments, a sample size of n ≥5 mice was used per experimental group.
Data exclusions	No data was excluded in this study.
Replication	Most of the in vitro experiments were repeated with at least three biological and/or technical replicates with similar results except for those specifically indicated in the figure legends. All attempts at replication were successful. The detail was indicated in the Methods section.
Randomization	For animal studies, Galectin-3 Knockout mice and control mice were randomly distributed into group and then earmarked by an independent researcher. db/db mice were grouped according to body weight, fasting blood glucose and insulin sensitivity before treatment.
Blinding	All experiments were performed in a non-blinded manner, because the experimental design was complicated, the researchers were limited, and blinding feasibility was poor.

Reporting for specific materials, systems and methods

We require information from authors about some types of materials, experimental systems and methods used in many studies. Here, indicate whether each material, system or method listed is relevant to your study. If you are not sure if a list item applies to your research, read the appropriate section before selecting a response.

Materials & experimental systems

n/a	Involved in the study	
☐	☒	Antibodies
☐	☒	Eukaryotic cell lines
☒	☐	Palaeontology and archaeology
☐	☒	Animals and other organisms
☒	☐	Clinical data
☒	☐	Dual use research of concern
☒	☐	Plants

Methods

n/a	Involved in the study	
☒	☐	ChIP-seq
☒	☐	Flow cytometry
☒	☐	MRI-based neuroimaging

Antibodies

Antibodies used	Western Blots: anti-flag Tag (MBL, #M185-3L, 1:10000), anti-GFP Tag (MBL, #598, 1:5000), anti-Phospho-Akt (S473) (Cell Signaling Technology, CST, #4060, 1:2000, D9E), anti-Akt (CST, #4691, 1:1000, C67E7), anti-beta Actin (Proteintech, #66009-l-lg, 1:5000, 2D4H5).<br>Immunofluorescence: anti-Galectin3 (Abcam, #ab2785, 1:100, A3A12), anti-CACNG1 (LSBio #LS-C805513, 1:200), anti-insulin (R&D Systems, #MAB1417, 182410, 1:200; Abcam, #ab7842, 1:100), anti-CD11c (CST, #97585, 1:100, D1V9Y), anti-glucagon (CST, #2760, 1:200), anti-F4/80 (1:100, Abcam, ab6640, CI:A3-1). Alexa Fluor 546 goat anti-Rabbit (Invitrogen, A11035, 1:200), Alexa Fluor 488 goat anti-mouse (Invitrogen, A11029, 1:200), Alexa Fluor 647 goat anti-rat (Invitrogen, A21247, 1:200), Alexa Fluor 647 goat anti-guinea pig (Invitrogen, A21450, 1:200), Alexa Fluor 594 goat anti-rat (Invitrogen, A-11007, 1:200).
Validation	Validation statements for all used antibodies are available at the websites of the commercial providers.<br>Anti-DDDDK-tag antibody, WB,IP,FCM,IC, ChIP, Co-IP and Other, (https://www.mbl-chinawide.cn/search012?keyword=M185); anti-GFP antibody, WB,IP,IC,IH,ChIP,Other, (https://www.mbl-chinawide.cn/search012?keyword=598); anti-Phospho-Akt (S473) (D9E) Rabbit antibody, human, mouse and rat, WB, IP, IHC, IF (https://www.cellsignal.cn/products/primary-antibodies/phospho-akt-ser473-d9e-xp-174-rabbit-mab/4060); anti-Akt (C67E7) rabbit antibody, human, mouse and rat, WB, IP, IHC and IF (https://www.cellsignal.cn/products/primary-antibodies/akt-pan-c67e7-rabbit-mab/4691); anti-beta Actin (2D4H5) antibody, human, mouse, rat, WB, IP, IHC, IF, FC, CoIP, ChIP, Cell treatment, ELISA, (https://www.ptglab.com/products/Pan-Actin-Antibody-66009-1-Ig.htm); anti-Galectin3 (A3A12) antibody, mouse and human, WB, ICC/IF and IHC-P (https://www.abcam.cn/products/primary-antibodies/galectin-3-antibody-a3a12-ab2785.html); anti-CACNG1 Rabbit antibody, human, mouse and Rat, IF, WB and Peptide-ELISA (https://www.lsbio.com/antibodies/cacng1-antibody-cacng-antibody-if-immunofluorescence-wb-western-ls-c805513/832078); anti-insulin (182410) antibody, human, mouse and bovine, ICC/IF and IHC (https://www.rndsystems.com/cn/products/human-mouse-bovine-insulin-antibody-182410_mab1417#product); anti-insulin antibody, mouse, human and Rat (https://www.abcam.cn/products/primary-antibodies/insulin-antibody-ab7842.html); anti-F4/80 (CI:A3-1) antibody, mouse, Flow Cyt, ICC/IF (https://www.abcam.cn/products/primary-antibodies/f480-antibody-cia3-1-macrophage-marker-ab6640.html); anti-CD11c (D1V9Y) Rabbit antibody, mouse, WB, IHC and IF (https://www.cellsignal.cn/products/primary-antibodies/cd11c-d1v9y-rabbit-mab/97585); anti-glucagon antibody, human, mouse and rat, IHC and IF (https://www.cellsignal.cn/products/primary-antibodies/glucagon-antibody/2760); Alexa Fluor 546 goat anti-Rabbit https://www.thermofisher.cn/cn/zh/antibody/product/Goat-anti-Rabbit-IgG-H-L-Highly-Cross-Adsorbed-Secondary-Antibody-Polyclonal/A-11035); Alexa Fluor 488 goat anti-mouse (https://www.thermofisher.cn/cn/zh/antibody/product/Goat-anti-Mouse-IgG-H-L-Highly-Cross-Adsorbed-Secondary-Antibody-Polyclonal/A-11029); Alexa Fluor 647 goat anti-rat (https://www.thermofisher.cn/cn/zh/antibody/product/Goat-anti-Rat-IgG-H-L-Cross-Adsorbed-Secondary-Antibody-Polyclonal/A-21247); Alexa Fluor 647 goat anti-guinea pig (https://www.thermofisher.cn/cn/zh/antibody/product/Goat-anti-Guinea-Pig-IgG-H-L-Highly-Cross-Adsorbed-Secondary-Antibody-Polyclonal/A-21450); Alexa Fluor 594 goat anti-mouse (https://www.thermofisher.cn/cn/zh/antibody/product/Goat-anti-Rat-IgG-H-L-Cross-Adsorbed-Secondary-Antibody-Polyclonal/A-11007).

Eukaryotic cell lines

Policy information about cell lines and Sex and Gender in Research

Cell line source(s)	MIN6 were obtained from Prof. Tao Xu in the Institute of Biophysics, Chinese Academy of Sciences. INS1 were obtained from ACCEGEN (ABC-TC232S). were purchased from the cell culture center of Peking Union Medical College. HepG2 cells were purchased from ATCC. FreeStyle 293F cells were provided by Prof. Wu in the Institute of Materia Medica, Chinese Academy of Medical Sciences and Peking Union Medical College.<br>The mice primary islets were obtained from males. The human primary islets were obtained from males and females.
Authentication	MIN6 and INS1 were authenticated by the cell morphology and function. HEK293T, HepG2 and FreeStyle 293F were authenticated by STR profiling. Primary islets were authenticated by staining with dithizone (DTZ).
Mycoplasma contamination	Cell lines were routinely tested for potential mycoplasma contamination by using commercial mycoplasma detection kits (Londa, LT07-418). All test were negative.
Commonly misidentified lines<br>(See ICLAC register)	There were no commonly misidentified lines in our study.

Animals and other research organisms

Policy information about studies involving animals; ARRIVE guidelines recommended for reporting animal research, and Sex and Gender in Research

Laboratory animals	Gal3-/- mice were kindly provided by Dr. Jerrold M. Olefsky from UC San Diego. db/+ mice were provided by Dr. Huang from Wenzhou Medical University. Gal3+/- mice were acquired by breeding Gal3-/- mice with WT C57BL/6J mice. WT, Gal3+/- and Gal3-/- littermates were produced by crossing Gal3+/- mice together. Gal3+/- db/+ mice were acquired by breeding Gal3-/- mice with db/+ mice. Gal3-/- db/db and db/db mice were produced by crossing Gal3+/- db/+ mice together. Ins1 cre-GCaMP6f mice were provided by Dr. Liangyi Chen's lab at Peking University. Ins1 cre-GCaMP6f mice were generated by crossbreeding Ins1(Cre) mice54 (Jackson Laboratories, strain #026801) and GCaMP6f fl/fl lines (Jackson Laboratories, strain # 029626) to obtain β-cell specific GCaMP6f, a protein encoding a Ca2+ indicator with green fluorescence. Ten- to 14-week-old male Ins1-GCaMP6f mice were used for the islet β-cell calcium imaging experiments. Cacng1 global KO mice (background C57BL/6JGpt, strain #T029821) and Cacng1fl/fl mice (background C57BL/6JGpt, strain #T020460) were purchased from Gempharmatech Co., Ltd. β-Cell-specific GCaMP6f-Cacng1βKO mice were acquired by breeding Cacng1fl/fl-GCaMP6ffl/fl mice with Cacng1fl/fl Ins1-cre+/- GCaMP6ffl/fl mice. C57BL/6JGpt-Lyz2em1Cin(iCre)/Gpt mice were purchased from Gempharmatech Co., Ltd (background C57BL/6JGpt, strain #T003822). ZK-003CKO (Gal3f/f) mice were obtained from BIOCYTOGEN. Macrophage specific Gal3 knockout mice (Gal3f/+-lyz Cre and Gal3f/f-lyz Cre) were acquired by breeding Gal3f/f mice and Lyz2-icre mice.

	Eight-week-old male C57BL6/J (WT) mice and Gal3-/- and Gal3+/- mice were fed a high-fat diet (HFD, 60% of kcal from fat; D12492, Research Diets) for 8-16 weeks (Macrophage specific Gal3 knockout mice fed HFD for 4 weeks). Plasma Gal3 levels were measured by an ELISA from Aviscera Bioscience. Plasma insulin levels were measured with ELISA kits from ALPCO.<br>Male mice were used in this study. All db/db mice were BKS background.
Wild animals	No wild animals were used in the study
Reporting on sex	It is much easier to induce obesity in male mice through HFD feeding. So male mice were hosted for HFD-fed induced obese mice model in our experiments
Field-collected samples	No field collected samples were used in the study.
Ethics oversight	All experiments using animals were performed in accordance with protocols approved by the Animal Experimentation Ethics Committee of the Chinese Academy of Medical Sciences, and all procedures were conducted in accordance with the guidelines of the Institutional Animal Care and Use Committees of the Chinese Academy of Medical Sciences. All animal procedures were consistent with the ARRIVE guidelines.

Note that full information on the approval of the study protocol must also be provided in the manuscript.

Plants

Seed stocks	does not use plants
Novel plant genotypes	does not use plants
Authentication	does not use plants
